# The *Phosphofructokinase* and *Pyruvate Kinase* Genes in *Apis andreniformis* and *Apis cerana indica*: Exon Intron Organisation and Evolution

**DOI:** 10.21315/tlsr2019.30.1.6

**Published:** 2019-01-31

**Authors:** Nurul I. Shullia, Rika Raffiudin, Berry Juliandi

**Affiliations:** Department of Biology, Faculty of Mathematics and Natural Sciences, Bogor Agricultural University, Dramaga Campus, Bogor 16680, Indonesia

**Keywords:** *Apis cerana indica*, *Apis andreniformis*, Phosphofructokinase Gene, Pyruvate Kinase Gene, Exon Gain and Loss

## Abstract

Genes related to carbohydrate metabolism have evolved rapidly in eusocial bees, including honey bees. However, the characterisation of carbohydrate metabolism genes has not been reported in *Apis andreniformis* or *Apis cerana indica*. This study aimed to characterise *phosphofructokinase* (PFK) and *pyruvate kinase* (PK) genes in these honey bee species and to analyse the evolution of the genus *Apis* using these genes. This study found the first data regarding *A. andreniformis* PFK and PK-like nucleotide sequences. A BLAST-n algorithm-based search showed that *A. andreniformis* and *A. c. indica* PFK and PK genes were homologous with those of *Apis florea* and *Apis cerana cerana* from Korea, respectively. Multiple alignments of PFKs from five *Apis* species showed many exon gains and losses, but only one among the PKs. Thus, the exon–intron organisation of the PK genes may be more conserved compare with that of the PFKs. Another evolutionary pattern indicated that more nucleotide substitutions occurred in *Apis*’ PK than PFK genes. Deduced PFK amino acid sequences revealed a PFK consensus pattern of 19 amino acids, while the deduced PK amino acid sequences were predicted to have barrel and alpha/beta domains. Based on these two metabolism-related genes, The Neighbour-joining and Maximum likelihood phylogenetic trees are congruent and revealed that the *A. andreniformis* and *A. florea* group were in the basal position. *Apis mellifera*, *A. cerana*, and *Apis dorsata* formed a monophyletic clade, although the positions of *A. mellifera* and *A. dorsata* were different in the nucleotide- and amino acid-based phylogenetic trees.

## INTRODUCTION

Honey bees use carbohydrates as the main fuel for flight and produce modified stored sugar (honey) to maintain the optimal hive temperature (Fischman *et al*. 2011). Molecular data has indicated that carbohydrate metabolism-related genes are among the most rapidly evolving genes in eusocial bees, including honey bees (Woodard *et al*. 2011). *Phosphofructokinase* (PFK; EC 2.7.1.11) plays a key regulatory role in the glycolytic pathway. It catalyses the reaction of fructose 6-phosphate using ATP to generate 1,6-diphosphate and ADP (Voet & Voet 1995). *Pyruvate kinase* (PK; EC 2.7.1.40) is involved in glycolytic flux and catalyses the reaction of pyruvate phosphoenol to generate ATP and pyruvate by transferring the phosphate group to ADP (Voet & Voet 1995).

The first eukaryotic PFK sequence was characterised in cloned rabbit muscle and its 17-kb length was split into 22 exons, encoding 780 amino acids (Lee *et al*. 1987). The exon–intron organisation was the same among human liver (Elson *et al*. 1990), human muscle (Vaisanen *et al*. 1992) and mouse liver (Rongnoparut *et al*. 1991) PFKs. In insects, the PFK gene has been characterised in *Drosophila melanogaster* and spans 6.5 kb, which is split into 8 exons and encodes 787 amino acids. The amino acid sequence of *D. melanogaster* PFK showed a 50.9% identity with the human muscle PFK (Currie & Sullivan 1994).

PK genes have also been characterised as 20 kb in rat muscle (Takenaka *et al*. 1989), and 32 kb in human muscle, consisting of 12 exons and 11 introns (Takenaka *et al*. 1991), whereas chicken PK has at least 10 introns (Lonberg & Gilbert 1985). Complementary DNA cloning of the PK gene in *D. melanogaster* revealed a 1,602-bp coding region split into four exons encoding a predicted 533 amino acids (Chien *et al*. 1999).

Database entries from GenBank showed that the PFK gene in the genus *Apis* has different exon numbers in different species. The whole genome of *A. mellifera* from NCBI showed that *ATP-dependent 6-phosphofructokinase* has 13 exons (GenBank NC_007079). However, this gene in the *A. cerana cerana* strain from Korea (GenBank NW_016019786) has 7 isoforms and 24 exons. The giant honey bee *Apis dorsata* (GenBank NW_006263741) has 7 isoforms and 22 exons, and the dwarf honey bee *Apis florea* (GenBank NW_003790158) has 14 exons. However, almost all of the *Apis* PK genes have similar exon–intron organisations. GenBank database entries showed that *A. dorsata* (GenBank NW_006263478), *A. florea* (GenBank NW_003790664), and *A. c. cerana* (GenBank NW_016019308) predicted PK (PK-like) genes have 8 exons, while *A. mellifera* (GenBank NC_007073) has 2 isoforms and 10 exons.

Sequences that have genetic variants are invaluable in documenting evolutionary history. Honey bee phylogenetic studies have been performed based on molecular data from mitochondrial genes, such as cytochrome c oxidase subunit I (*COI*) (Tanaka *et al*. 2001), cytochrome c oxidase subunit II (*COII*), rRNA gene for the large ribosomal subunit *rrnL*, and *NADH dehydrogenase subunit 2* (*nad2*), or from nuclear genes, such as *inositol 1,4,5-triphosphate receptor* (*itpr*) (Raffiudin & Crozier 2007) and the *elongation factor 1-alpha* (*EF1-α*) intron (Arias & Sheppard 2005). The position of dwarf bees (the *A. andreniformis* and *A. florea* group) is almost at the tree’s base, and the giant (the *A. dorsata* and *Apis laboriosa* group) and medium-sized bees (the *A. mellifera* and *A. cerana* group) form a monophyletic clade. A phylogenetic study based on several genes, including carbohydrate metabolism-related genes, has been reported for eusocial bees (Woodard *et al*. 2011), but the evolution of PFK and PK genes has not been explored in honey bees at the species level.

Indonesia has the most diverse honey bee population in the world, with five, *A. dorsata*, *A. cerana* (Ruttner 1988), *A. andreniformis* (Wu & Kuang 1987), *Apis koschevnikovi* (Tingek *et al*. 1988), and *Apis nigrocincta* (Hadisoesilo *et al*. 1995), of nine species of honey bee being native to Indonesia. *A. cerana* is distributed in the most of the Indonesian islands. Four subspecies of *A. cerana* are distributed in the old world, and *A. c. indica* is established in Indonesia (Ruttner 1988). The PFK and PK genes of the dwarf honey bee *A. florea* (Lowe & Eddy 1997) and the other subspecies *A. c. cerana* (Park *et al*. 2015) have been submitted as GenBank database entries, but those of *A. andreniformis* and *A. c. indica* from Indonesia have not been reported. This study aimed to characterise PFK and PK genes in *A. andreniformis* and *A. c. indica* and also to analyse the evolution of honey bees based on these genes.

## MATERIALS AND METHODS

### Samples and DNA Extraction, Amplification and Sequencing

*Apis andreniformis* was collected from Padang Pariaman, West Sumatra and *A. c. indica* was collected from Bogor, West Java, Indonesia. Total DNA was extracted from the thoraxes using a standard phenol–chloroform extraction method and ethanol precipitation (Sambrook *et al*. 1989), with minor modifications (Raffiudin & Crozier 2007).

The partial regions of PFK and PK-like gene primers were designed manually from *A. mellifera* (GenBank NC_007079, NC_007073), *A. dorsata* (GenBank NW_006263741, NW_006263478), and *A. florea* (GenBank NW_003790158, NW_003790664) genomic sequences. Due to an obstacle in primer design involving the 1,099 bp of Intron 3 in the *A. mellifera* PFK gene, the targeted gene was divided into two regions, Part A (exons 1–3) and Part B (exon 4–7) (Table 1). The PCR conditions were as follows: initial denaturing at 95°C for 3 min, 30 cycles of 95°C for 1 min, 48°C–53°C for 30 s, and 72°C for 1 min, followed by a final extension at 72°C for 2 min. PCR products were electrophoreses in 1.5% agarose gel and stained using Diamond Nucleic Acid Dye (Promega, Madison, WI, USA). The PCR products were sequenced by a company sequencing service (First BASE, Selangor, Malaysia).

### Gene Structure, Motif and Phylogenetic Analyses

The sequences of the PFK and PK-like genes from *A. andreniformis* and *A. c. indica* were aligned with homologues from *Apis* database entries in GenBank identified using a BLAST-n algorithm-based search of the nucleotide collection (nt/nt) (http://blast.ncbi.nlm.nih.gov/Blast.cgi). Based on homology analyses of the DNA coding regions and genomics, the closest related species to *A. andreniformis* and *A. c. indica* were aligned using ClustalX 2 (Larkin *et al*. 2007) and were used to determine the exon–intron organisation. Protein motifs and families of the putative amino acid sequences were explored using PROSITE (http://prosite.expasy.org/) and Pfam (http://pfam.sanger.ac.uk/), respectively. The number of substitutions and pairwise distances of *Apis* PFK and PK-like nucleotide sequences were analysed using MEGA6 (Tamura *et al*. 2013). The obtained sequences, combined with other *Apidae* sequences in GenBank, were chosen for phylogenetic analysis (Table 2). The nucleotide-based phylogenetic trees were constructed using the Neighbour-joining (NJ) with Tamura-Nei Model and Maximum likelihood (ML) method with suggested Best Model Tamura-Nei implemented in MEGA6 with 1,000 bootstrap replicates (Tamura *et al*. 2013). The amino acid-based phylogenetic trees were also constructed using NJ method with Poisson model and ML method with suggested Best model cpREV+G implemented in MEGA6 with 1,000 bootstrap replicates (Tamura *et al*. 2013).

## RESULTS

### Characterisation of Partial *PFK* and *PK-like* Genes

**Figure 1:**
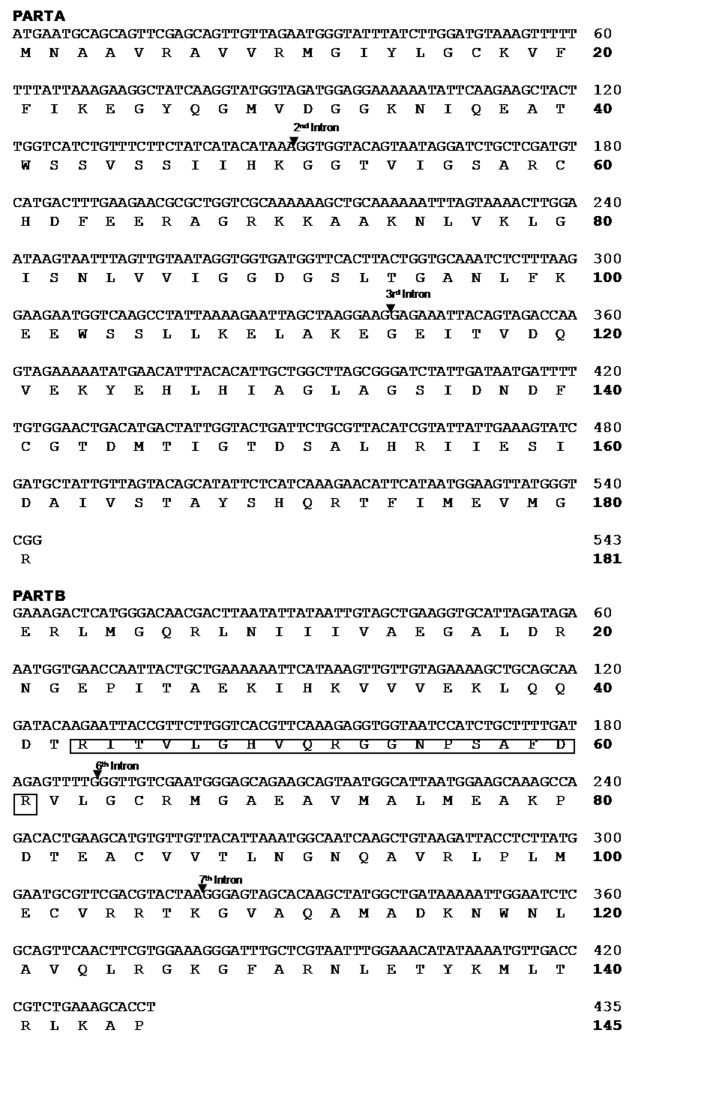
Nucleotide and deduced amino acid sequences of the *Apis andreniformis* PFK gene. The numbering on the right indicates the position of the last nucleotide or amino acid sequence in each line. The PFK signature based on the PROSITE analysis is boxed. Arrows indicate inserted introns.

**Figure 2:**
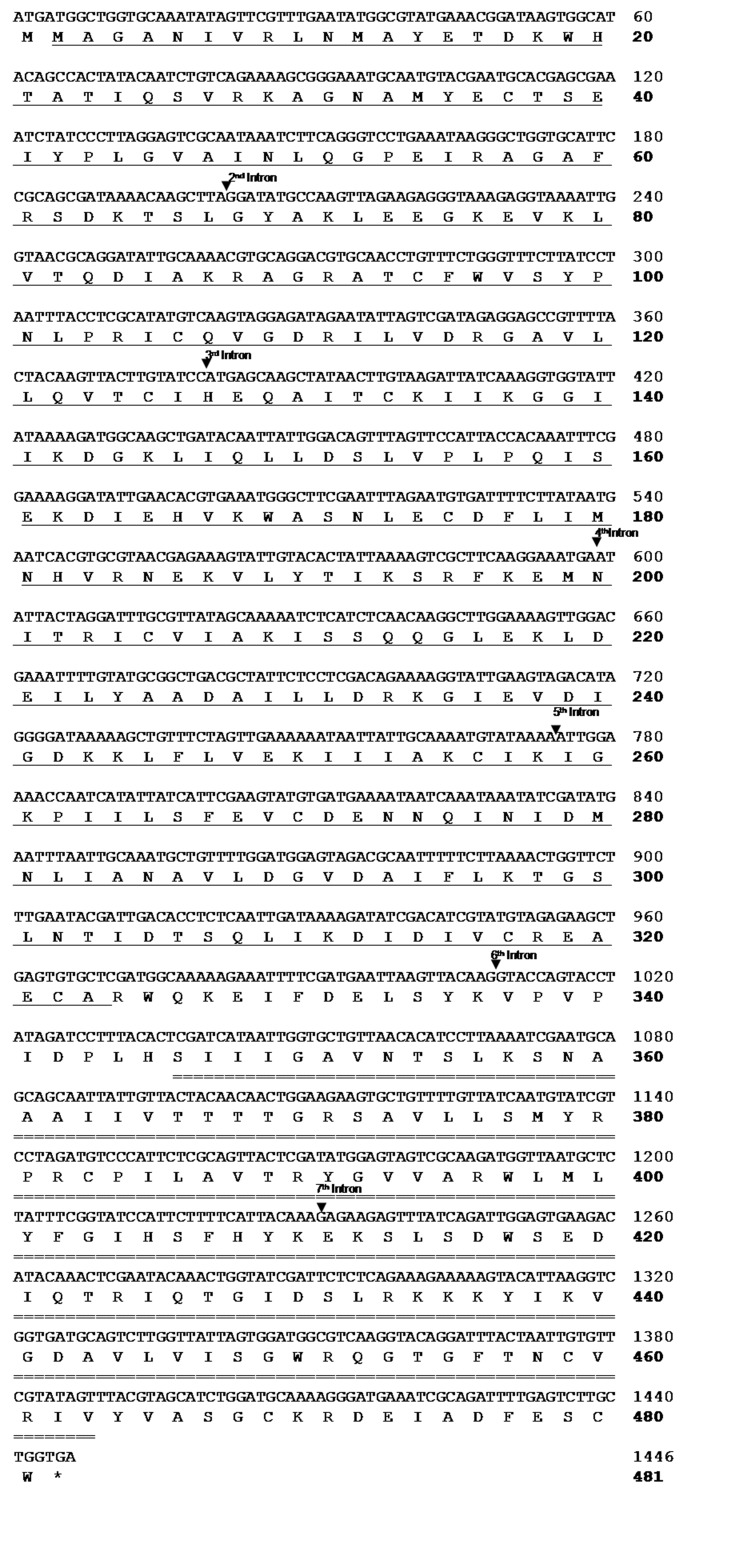
Nucleotide and deduced amino acid sequences of the *Apis andreniformis* PK-like gene. The numbering on the right indicates the position of the last nucleotide or amino acid in each line. PK barrel and PK alpha/beta domains are indicated by single and double underlines, respectively. Arrows indicate inserted introns. Asterisk (*) indicates the stop codon.

This study successfully amplified the targeted PFK and PK-like genes in *A. andreniformis* from Padang Pariaman, West Sumatra and *A. c. indica* from Bogor (DDBJ LC318660, LC318759-63). BLAST-n algorithm-based searches using the nucleotide collection (nr/nt) database showed that *A. andreniformis* is closely related to *A. florea*, with 100% (GenBank XM_012485123.1) and 95% (GenBank XM_012487945.1) identities for PFK and PK-like genes. Based on this homology and previous morphology (Alexander 1991), a combined behavioural and molecular phylogenetic study (Raffiudin & Crozier 2007) revealed that *A. andreniformis* and *A. florea* are sister species. Thus, *A. florea* PFK and PK coding regions and genomes were used to determine *A. andreniformis*’ exon–intron organisation. There were six exons (Exons 2, 3, 4, 6, 7, and 8) and four introns (Introns 2, 3, 6 and 7) of *A. andreniformis* in the A and B PFK sequences (Fig. 1), while the *A. andreniformis* partial PK-like sequence had seven exons (Exons 2–8) and six introns (Introns 2–7) (Fig. 2). The putative exon regions of *A. andreniformis*’ PFK parts A and B, and PK-like sequences revealed 543, 435 and 1,446 bp, respectively. Translations of *A. andreniformis* part A, PFK part B, and PK-like exon regions revealed 181, 151, and 482 putative amino acids, respectively.

There was also a high similarity between *A. c. indica* from Bogor, Indonesia and *A. cerana* from Korea, with 100% (GenBank XM_017065124.1) and 99% (GenBank XM_017058664.1) identities for the PFK and PK-like genes, respectively. Using the sequence of the *A. cerana* strain from Korea revealed that *A. c. indica* PFK parts A and B contained seven exons (Exons 6–12) and five introns (Intron 6, 7, 9, 10 and 11). The exon region of *A. c. indica* PFK parts A and B cover 537 and 510 bp, respectively. The partial PK-like sequence of *A. c. indica* s consists of seven exons (Exons 2–8) and six introns (Exons 2–7), which corresponds to 1,380 bp. The complete translations of *A. c. indica*’ PFK parts A and B, and the PK-like sequence encompassed 179, 170 and 490 putative amino acids, respectively.

**Figure 3:**
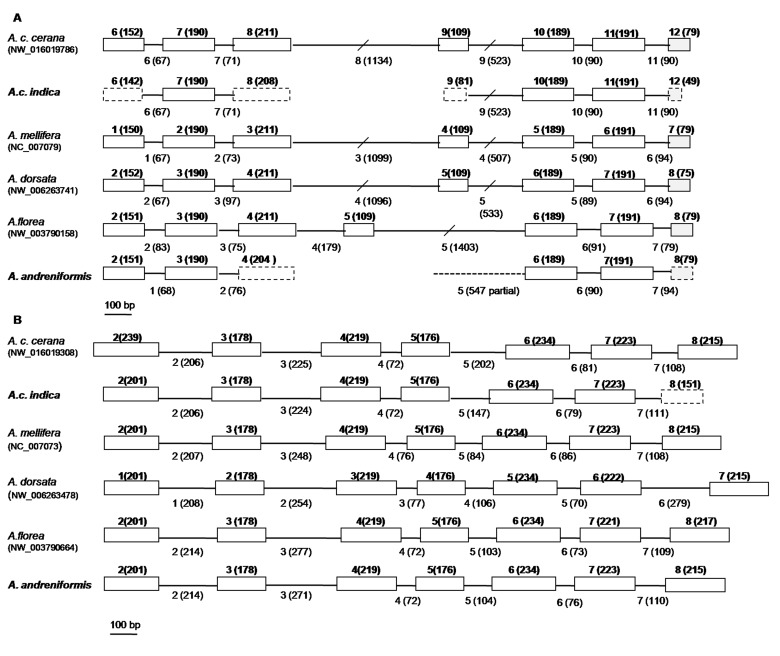
Schematic representations of exon and intron structures of PFK (A) and PK-like (B) genes from the genus *Apis* indicate a phenomenon of exon gain and loss. Boxes and lines indicate exons and introns, respectively. Numbers above the boxes and numbers in brackets indicate the exon numbers and exon lengths, respectively. Intron numbers and intron lengths are below each line.

Schematic representations of the PFK and PK-like exon–intron organisation in the genus *Apis* (Fig. 3) showed that the former had more variation than the latter in the genus *Apis*, even though their exon lengths are the same. Exons 6–12 in the *A. c. indica* PFK gene had similar sequences to Exons 1–7 of *A. mellifera* (GenBank NC_007079). The sequence of Exon 9 in *A. cerana* (GenBank NW_016019786) or Exon 4 in *A. mellifera* PFK gene was part of Intron 5 in the dwarf honey bee (GenBank NW_003790158). Thus, there is only one exon gain and one PK-like gene loss among these five species in the genus *Apis*.

All exon–intron boundaries in the PFK and PK-like genes were confirmed using GT–AG rules (Tables 3 and 4). Although the ranges of PFK and PK-like intron lengths were different in *A. andreniformis* and A*. c. indica*, the homologous introns had the same intron phase. Intron 5 of the *A. andreniformis* PFK gene was incomplete because the region was unamplified. The differences in intron lengths between *A. andreniformis* and *A. c. indica* were caused by base insertions and deletions.

### Motifs of Partial *PFK* and *PK-like* Genes

**Figure 4:**
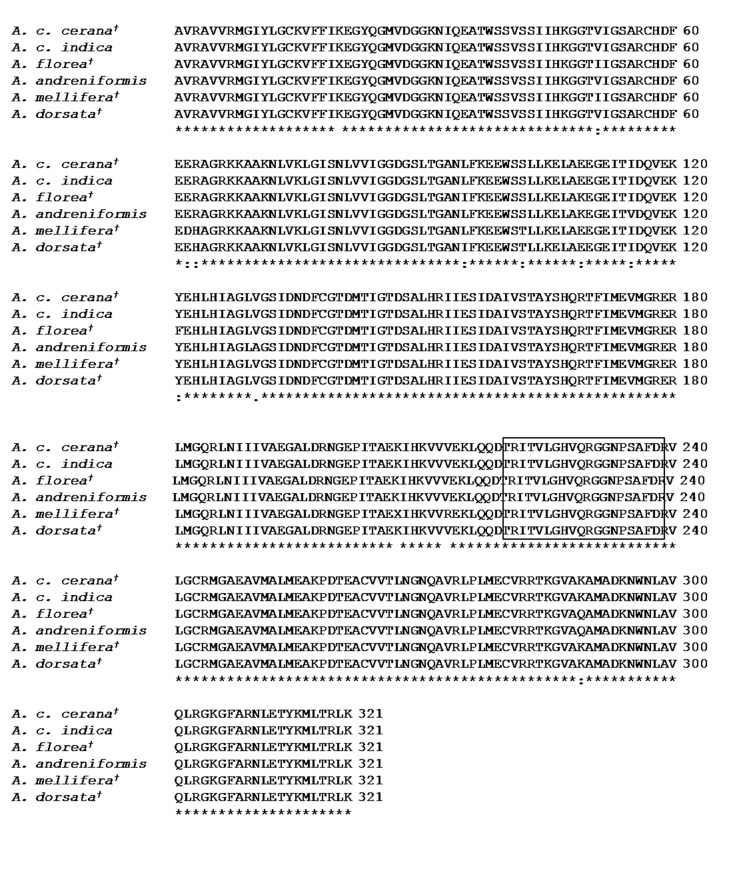
Multiple alignment of *Apis* PFK amino acid sequences. The number indicates the position of the last amino acid in the line. The PFK signature based on the PROSITE analysis is boxed. Dagger (†) indicates GenBank Accession numbers found in Table 2. Asterisk (*) indicates conserved amino acid sequences.

Motif searches using PROSITE (http://prosite.expasy.org/) showed a consensus pattern, [RK]-x(4)-[GAS]-H-x-[QL]-[QR]-[GS]-[GF]-x(5)-[DE]-[RL] PFK (PS00433), in both *A. andreniformis* and *A. c. indica* partial PFK sequences. This study also found a conserved PFK-related consensus pattern in the genus *Apis* (Fig. 4). Analysis of the protein family using Pfam indicated that *A. andreniformis* and *A. c. indica* partial PK-like amino acid sequences formed a pattern of a pyruvate kinase barrel domain at amino acids 2–323 and a pyruvate kinase alpha/beta domain format amino acids 345–463.

### Phylogenies of The Genus *Apis*’ *PFK* and *PK*-*Like* Genes

**Figure 5:**
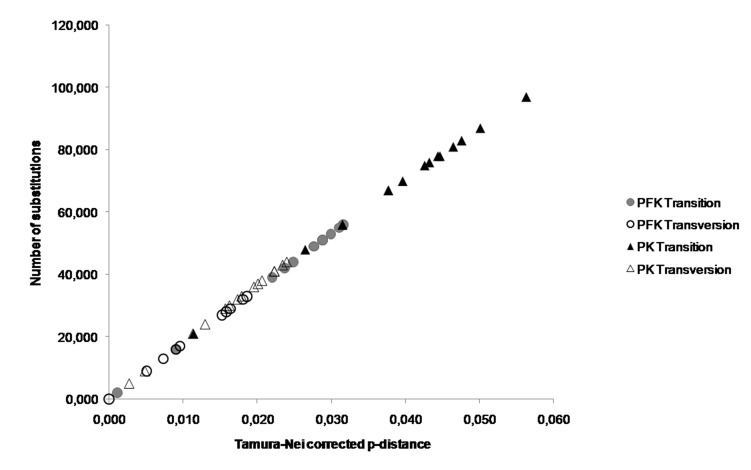
The relative transition and transversion rates of PK-like gene are higher than PFK gene in *Apis*.

**Figure 6:**
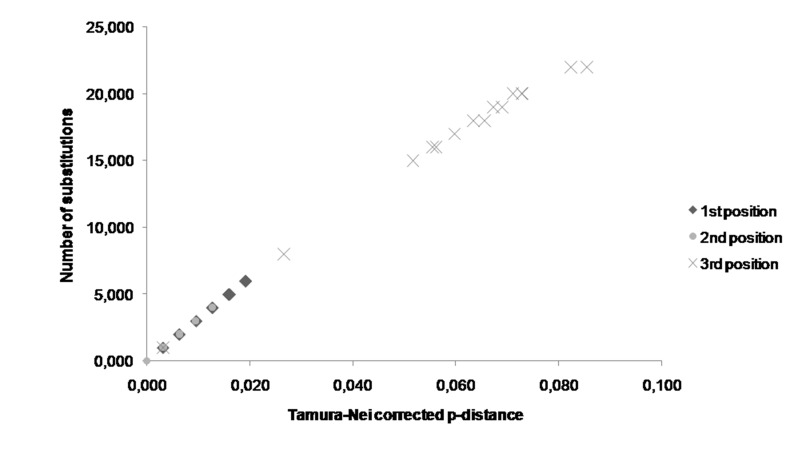
The difference of PFK exon substitution numbers for each codon position in *Apis*.

**Figure 7:**
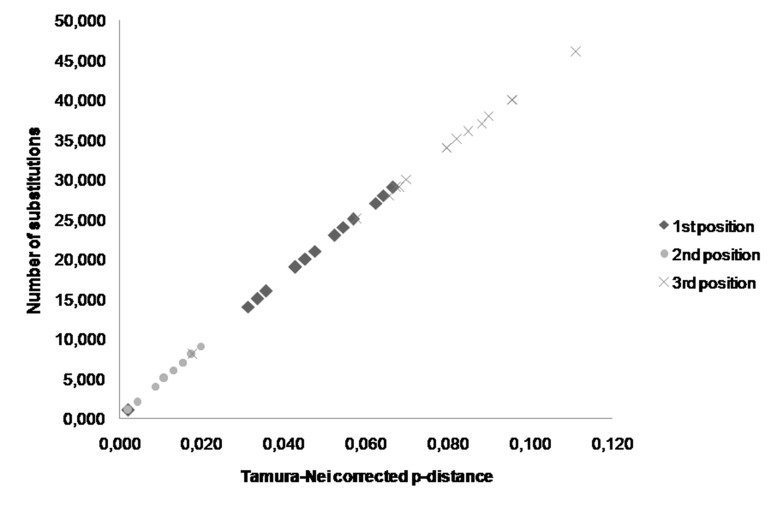
The difference of PK-like exon substitution numbers for each codon position in *Apis*.

The comparisons between the number of substitutions and the Tamura–Nei corrected p-distances showed that transitions occurred more often than transversions in the PFK and PK-like genes of these five *Apis* species. The p-distances corrected by Tamura-Nei were greater in the PK-like gene than in the PFK gene (Fig. 5). Analyses of pairwise comparisons revealed that the third codon substitution number (transition and transversion) was the highest in both PFK and PK-like gene sequences (Figs. 6 and 7). The range of the number substitutions in the exon regions in *Apis* PK-like gene sequences was wider than in the PFK gene.

**Figure 8:**
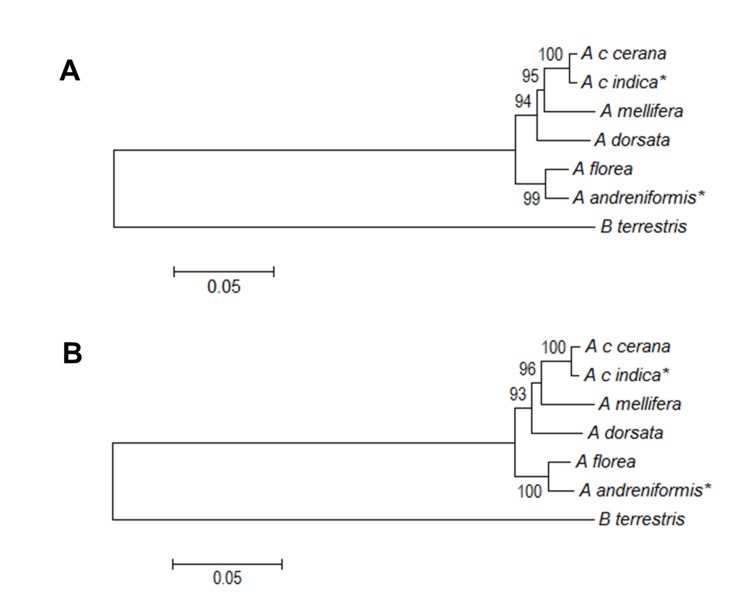
Nucleotide sequence-based phylogenetic tree of combined PFK and PK-like genes in the genera *Apis* and *Bombus* using (A) Neighbour joining and (B) Maximum likelihood methods with 1,000 bootstraps replication. Asterisk (*) indicates species used in this study.

**Figure 9:**
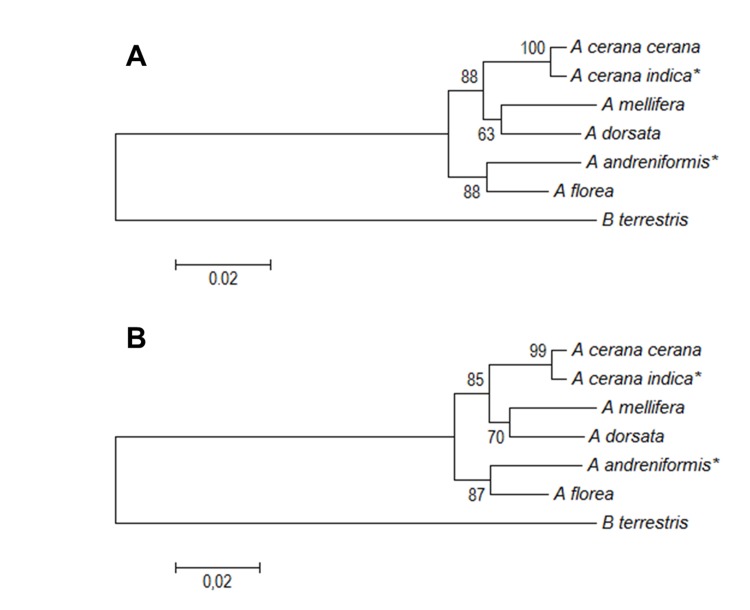
Amino acid sequence-based phylogenetic tree of combined PFK and PK-like genes in the genera *Apis* and *Bombus* using (A) Neighbour joining and (B) Maximum likelihood methods with 1,000 bootstraps replication. Asterisk (*) indicates species used in this study.

Using a combination of PFK and PK-like nucleotide (Fig. 8) and amino acid (Fig. 9) sequences in the genus *Apis* and out group, this study found two topologies based on nucleotide sequence and amino acid phylogenetic tree. The topology of both phylogenetic trees based on NJ and ML for nucleotide and amino acid sequences are the same. All of the trees supported the dwarf honey bee’s (*A. florea and A. andreniformis*) basal position. The nucleotide-based topology showed that the giant honey bee *A. dorsata* is the sister clade of the medium honey bee (*A. c. cerana*, *A c. indica*, and *A. mellifera*) (Fig. 8 A–B), but the amino acid-based topology placed *A. mellifera* and *A. dorsata* in a separate clade (Fig. 9 A–B).

## DISCUSSION

### Motifs in *PFK* and *PK* Genes in *Apis*

This study aimed to characterise PFK and PK-like genes, which are key regulatory enzymes in glycolysis and control the flux through this pathway (Voet & Voet 1995). We studied these two genes in the native Indonesian honey bee *A. andreniformis* and the widely distributed *A. c. indica*. This is the first data regarding *A. andreniformis* PFK and PK-like nucleotide sequences. Analyses of deduced *A. andreniformis* and *A. c. indica* PK-like amino acids determined that these sequences have barrel and alpha/beta domains. Muirhead (1986) found that the cat PK gene in muscle consists of four domains: N-terminal, A (A1 and A2), B, and C. The complementary DNA of the *Drosophila* PK gene also has four domains and a conserved amino acid in the active site (Chien *et al*. 1999).

A PROSITE analysis determined that the PFK sequences contain the [RK]-x(4)-[GAS]-H-x-[QL]-[QR]-[GS]-[GF]-x(5)-[DE]-[RL] PFK (PS00433) consensus pattern. This corroborates our investigation of the *Apis* PFK gene in which a multiple alignment revealed the consensus pattern of RITVLGHVQRGGNPSAFDR. The R or K amino acid, and the H and Q or R amino acids are important because of their involvement in fructose-6-phosphate binding (http://prosite.expasy.org/). The R and H amino acids were also found in the N- and C-halves of two adjacent subunits in the rabbit muscle PFK and defined the binding-site of fructose-6-phosphate (Poorman *et al*. 1984).

### Exon Gain and Loss in The *PFK* and *PK* Genes of *Apis*

The NCBI database entries for PFK genes in the genus *Apis* showed variations in number of exons, with 13 exons in *A. mellifera* (GenBank NC_007079) and up to 24 exons in *A. c. cerana* (Genbank NW_016019786). This variation indicated a phenomenon of exon gain and loss in the PFK gene. This lead to the sequence of Exon 9 from *A. c. indica* and *A. c. cerana* or Exon 4 from the *A. mellifera* PFK gene being part of Intron 5 in *A. florea* and *A. andreniformis*.

Like the PFK exon number among the genus *Apis*, human (Vaisanen *et al*. 1992) and rabbit PFK genes in muscle have up to 22 exons (Lee *et al*. 1987). However, the PFK gene of *D. melanogaster* that contains 6.5 kb, only has half the *Apis* PFK exon number (eight exons and seven introns) (Currie & Sullivan 1994). This suggests that the PFK gene in the genus *Apis* was more evolved than that of *Drosophila*. The losses of exons might be caused by frame shift mutations or splice junctions that resulted in intron sliding (Currie & Sullivan 1994).

*A. andreniformis* and *A. c. indica* have eight exons in their PK-like genes and show similar exon-intron organisations. However, the *Drosophila* PK gene has only half the exon number compared with *Apis* (Chien *et al*. 1999). Although the *Apis* PK-like genes have more similar exon-intron organisations than the PFK genes, another study revealed that *A. mellifera* and *Drosophila* PFK genes had a 1:1 orthology, while the PK gene had a 2:6 orthology (Kunieda *et al*. 2006). The greater diversity level of the PK-like gene may be a result of its position at the end of glycolysis pathway, before pyruvate enters the citrate cycle or other pathways (Kunieda *et al*. 2006). The PFK gene evolved by gene duplications and the amino acid sequence is highly homologous between prokaryotes and mammals (Poorman *et al*. 1984). The presence of orthologous PK-like genes in the genus *Apis* might be caused by the high nucleotide substitution rate in the PK-like gene compared with that of the PFK gene.

### The Evolution of *Apis PFK* and *PK* Genes

Here, the PK-like gene had more substitutions than the PFK gene. Thus, we analysed the evolution of the genus *Apis* based on the combined data regarding PFK and PK-like genes. The resulting Neighbour-joining phylogenetic tree of the honey bee that confirmed by Maximum likelihood phylogenetic showed that the dwarf honey bee (the *A. andreniformis* and *A. florea* group) was always in basal position. The tree also grouped the medium-sized honey bee (the *A. cerana* and *A. mellifera* group) and giant honey bee (*A. dorsata*) into a monophyletic clade, but *A. mellifera* and *A. dorsata* formed two topologies. The first topology built from combined PFK and PK-like nucleotide sequences was congruent with phylogenetic tree based on the molecular sequences of five genes and the behavioural states (Raffiudin & Crozier 2007). Almost all of the phylogenetic trees based on the molecular data grouped honey bees into three major clusters based on body size: giant bees, dwarf bees, and medium bees. Molecular-based honey bee phylogenetic trees were also congruent with the morphology-based phylogenetic tree (Alexander 1991). This indicated that nucleotide variations in intron regions also had roles in building the phylogenetic tree. The substitution rates in PFK and PK-like genes were greater in the third and first codons, respectively, than in the second codon. This result supported the finding that transitions in *16S rRNA*, *COI*, and *COII* genes were more common than transversions in the genus *Apis* (Tanaka *et al*. 2001). In a future study, an analysis of the cDNAs of these genes in the honey bee is needed to fully analyse the phenomenon of exon gain and loss in *Apis* evolution.

## CONCLUSION

Characterisations of *A. andreniformis* and *A. c. indica* PFK and PK-like genes revealed that they have same exon–intron organisation as *A. florea* and *A. c. cerana* from Korea, respectively. Moreover, multiple alignments of these genes among five *Apis* species revealed that exon gain and loss occurred more often in PFK than in PK-like genes, even though the nucleotide substitution rate in the former was higher than in the latter. The nucleotide-based phylogenetic tree generated from the combination of data on the two carbohydrate metabolism-related genes was congruent with molecular and morphological phylogenetic trees, and clustered *A. mellifera* and *A. cerana* groups with *A. dorsata* to form a monophyletic clade, while the *A. florea* and *A. andreniformis* group was basal.

## Figures and Tables

**Table 1:** PFK and PK-like gene primers designed based on the *Apis mellifera* whole genome.

No.	Gene/Exon*	Primer names	Primer Nucleotide (5′–3′)	*A. mellifera* Acc number
1	Part A PFK (Exons 1–3)	Am_PFK1_F	**GGAATGAATGCAGCAGTTCGAG**	GenBank NC_007079
		Am_PFK1_R	**CAATGCCGACCCATAACTTCCA**	
2	Part B PFK (Exons 4–5)	Am_PFK3_F	**GCAGCCATATGTTCTGAAGCTG**	
		Am_PFK3_R	**ACCACCTCTTTGAACATGACCA**	
3	Part B PFK (Exons 5–7)	Am_PFK4_F	**GAAAGACTTATGGGACAACGACT**	
		Am_PFK4_R	**ATAGGTGCTTTCAGACGGGTCA**	
4	PK-like (Exons 1–2)	Amel_PK1_F	**TGGCCGGTGCAAATATAGTTCG**	GenBank NC_007073
		Amel_PK1_R	**AACTTGTAATAAAACGGCTCCTC**	
5	PK-like (Exons 2–4)	Amel_PK2_F	**AGGACGTGCAAACTGTTTCTGG**	
		Amel_PK2_R	**TGTCGAGGAGAATAGCGTCAG**	
6	PK-*like* (Exons 4–6)	Amel_PK3ex_F	**GATTACTAGAATTTGCGTTATAGC**	
		Amel_PK3in_F	**ATCTCGTCTCAACAAGGTTTGGA**	
		Amel_PK3_R	**GCGAGAATGGGACATCTAGGAC**	
7	PK-*like* (Exon 7)	Amel_PK4_F	**CACTCGATCATAATTGGTGGTGT**	
		Amel_PK4in_R	**GGGACTTGATCCTTTCTGCATC**	
		Amel_PK4ex_R	**TCACCAGCAAGACTCAAAATCTG**	

**Table 2:** Species used for phylogenetic analysis from current research and GenBank.

No.	Species	Abbreviation	Accession number		References

			PFK	PK-like	
	In group		DDBJ		
			
1	*A. c. indica*	Ace	LC318760-61	LC318660	current research
2	*A. andreniformis*	Aan	LC318762-63	LC318759	current research
			
			GenBank		
			
3	*A. c. cerana*	Acc	NW_016019786	NW_016019308	(Park *et al*. 2016)
4	*A. mellifera*	Ame	NC_007079	NC_007073	(The Honeybee Genome Sequencing Consortium 2006)
5	*A. florea*	Afl	NW_003790158	NW_003790664	(Lowe and Eddy 1997)
6	*A. dorsata*	Ado	NW_006263741	NW_006263478	(Lowe and Eddy 1997)

	Out group				
7	*Bombus terrestris*	Bte	NC015771	NC015765	–

**Table 3:** Intron lengths in partial *A. andreniformis* PFK and PK-like genes.

Gene	Intron number	Intron length	5′ splice site (exon/intron)	3′ splice site (intron/exon)	Intron phase
PFK (Part A)	2	68	**AAA/****gt****atatattatg**	**attttacttt****ag****/GGT**	0
	3	76	**GAA,G/****gt****aaataaaa**	**gtttattt****ag****/GA,GAA**	1
PFK (Part B)	5	547	**NA**	**atataatttc****ag****/GAA**	NA
	6	90	**TTG/****gt****tagttattat**	**taataataat****ag****/GGT**	0
	7	79	**GGA,AA/****gt****atgtctt**	**atattttt****ag****/G,GGA**	2
PK-like	2	214	**TTA/****gt****acgatattaa**	**ttatatttac****ag****/GGA**	0
	3	271	**ATC,C/****gt****tagtttat**	**tcgatac****ag****/AT,GAG**	1
	4	72	**ATG,A/****gt****atgcgtat**	**tatttaa****ag****/AT,ATT**	1
	5	104	**AAA/****gt****aagtctatta**	**ttttttctcc****ag****/ATT**	0
	6	76	**AAG/****gt****agaaaaactt**	**ttataaaacc****ag****/GTA**	0
	7	110	**AAA,G/****gt****aaatatat**	**gtaattt****ag****/AG,AAG**	1

*Note:* NA = The ‘5 splice site and intron phase was not available due to the intron was not complete.

**Table 4:** Intron lengths in partial *A. c. indica* PFK and PK-like genes.

Gene	Intron number	Intron length	5′ splice site (exon/intron)	3′ splice site (intron/exon)	Intron phase
PFK (Part A)	6	67	**AAA/****gt****atgtattatg**	**attttaattt****ag****/GGT**	0
	7	71	**GAA,G/****gt****aagtaaaa**	**tttattt****ag****/GA,GAA**	1
PFK (Part B)	9	523	**CAG/****gt****tcgcaatttt**	**atataatttc****ag****/GAA**	0
	10	90	**TTG/****gt****tagttattat**	**taataataat****ag****/GGC**	0
	11	90	**GGA,AA/****gt****atgtctt**	**ttttagtt****ag****/G,GGA**	2
PK-like	2	206	**TTA/****gt****acgatattaa**	**ttatatttac****ag****/GGT**	0
	3	224	**ATC,C/****gt****tagttttt**	**tcaatac****ag****/AT,GAG**	1
	4	72	**ATG,A/****gt****atgcgtat**	**tatttaa****ag****/AT,ATT**	1
	5	147	**AAA/****gt****aagtttatta**	**ttttttctcc****ag****/ATG**	0
	6	79	**AAA/****gt****agaaaaactt**	**attcaaaacc****ag****/GTA**	0
	7	111	**AAA,G/****gt****aaatatat**	**ataattt****ag****/AG,GAA**	1
